# Antimicrobial prescription data in Danish national database validated against treatment records in organic pig farms and analysed for associations with lesions found at slaughter

**DOI:** 10.1186/s12917-019-1913-x

**Published:** 2019-06-27

**Authors:** Amanda Brinch Kruse, Charlotte Sonne Kristensen, Ulrik Lavlund, Helle Stege

**Affiliations:** 1 University of Copenhangen, Copenhagen, Denmark; 2 Danish Pig Research Centre, Copenhagen, Denmark; 3 Danish Veterinary and Food Administration, Copenhagen, Denmark

**Keywords:** Pig production, Organic, Antimicrobial use, Meat inspection, Prescription data, Primary data, Validation

## Abstract

**Background:**

Antimicrobial use (AMU) in livestock is a debated topic, mainly due to the risk of associated development of antimicrobial resistance. There is focus on reducing AMU in the Danish pig production, which accounts for the largest proportion of AMU for animals in Denmark. Due to special restriction on AMU in organic pig production, the AMU in organic pig production is lower than in conventional pig production. There is concern that reduced AMU could jeopardize animal health and welfare, if it reflects insufficient treatment of sick animals, which might be reflected by the prevalence and types of lesions found at meat inspection. However, little is known about the associations between AMU and meat inspection findings in pigs from organic farms. Furthermore, excess amount of antimicrobial product after a treatment cannot be re-prescribed in organic pig herds. The initial prescription is recorded in the national database VetStat, but the unused amount is not deducted leading to uncertainty when reporting AMU. The objectives of this study were to 1) describe AMU patterns based on prescription data for organic pig production and compare with those of the conventional pig production for year 2016, 2) study the associations between herd-level AMU prescription data and meat inspection data for organic pig herds and 3) validate herd-level AMU prescription data in VetStat against treatment records collected on-farm in organic Danish pig herds.

**Results and conclusions:**

Gastrointestinal indications account for the largest proportion of AMU in both organic pig herds (65 and 54% of treatment doses for weaners and finishers, respectively) and conventional pig herds (80 and 68% of doses for weaners and finishers, respectively). A larger proportion is prescribed for respiratory indications in organic than conventional weaners and arthropathic indications in finishers. No associations between AMU and meat inspection data were found. This needs further investigation as the prevalence of lesions at slaughter was slightly (non-significantly) higher in herds with no registered AMU than with AMU prescriptions. Only 8 out of 31 herds had recorded their AMU sufficiently detailed to compare, and using VetStat as a proxy for AMU led to 9–88% overestimation of the actual use in 7 out of these 8 herds and 120% underestimation in one herd.

## Background

Over the last decades there has been increasing concern regarding antimicrobial use (AMU) in food producing animals and development of antimicrobial resistance (AMR), due to the risk that AMR can spread from animals to the humans [19]. A prerequisite for reducing AMU is to have national surveillance systems for AMU, which are now established in many European countries (http://www.aacting.org/monitoring-systems/). In Denmark, the veterinary AMU at herd-level has been monitored in the database VetStat since 2000 [18]. Also, these data are frequently used in scientific studies and presented in international papers [8, 11, 14]. The primary focus in these studies has been on AMU in pig production, which accounts for 70% of the total AMU for animals in Denmark [7]. So far, the use of antimicrobials specifically in Danish organic pig herds has not been studied much, probably because the organic pig production only contribute with 0.9% of the total number of pigs slaughtered in Denmark [6, 12]. AMU in organic pig herds is lower than in the non-organic pig production. Thus, the organic pigs only account for 0.2% of the total AMU in Danish pigs (Vetstat data, unpublished). This might be because of a more restricted policy on AMU in organic pig herds.

There have been many initiatives to reduce AMU in Danish pig production in general, and the total AMU measured in kg active compound has declined by 28% over the last 10 years [7]. The Danish pig production has a low AMU compared to other countries with a similar intensive pig production [10]. Despite an even lower level of AMU in Danish organic pig herds, a continued focus on reducing AMU in pig production in Denmark might also affect the AMU in the organic pig production in the future. The general concern is that further reduction in AMU could jeopardize animal health and welfare due to lack of or insufficient treatment of sick pigs. Previous studies have shown that pigs raised in some Danish organic herds suffer from conditions that require antimicrobial treatment [2, 13]. However, the associations between AMU in organic herds and the prevalence of those diseases have not yet been studied at herd-level.

Prescription data from VetStat has previously been used as a proxy for AMU in conventional pigs. However, it might be challenging to do the same for organic pig production. According to national legislation, any excess antimicrobial product after a treatment course in organic herds cannot be stored on farm and re-prescribed for next treatment as in non-organic herds [3]. Therefore, organic farmers must discard excess amounts safely, e.g. by returning it to the pharmacy or the veterinarian. However, these discarded amounts are not registered in VetStat (i.e. they are not subtracted from original registration) why prescription data might not be a valid proxy for the actual use at herd-level for organic pig herds. So far, the extent of unused antimicrobials from organic herds has not yet been investigated.

Based on the presented issues and concerns related to AMU in organic pig production this study had three objectives:

1. Describe AMU patterns based on prescription data for organic pig production and compare with those of the conventional pig production for year 2016
2. Study the associations between herd-level AMU prescription data and meat inspection data from organic pig herds
3. Validate herd-level AMU prescription data in VetStat against treatment records collected on-farm in organic pig herds

## Results

The first objective of the study was to describe AMU patterns based on prescription data for organic pig production and compare these with those of the conventional pig production. In Denmark, there were 122 organic pig herds recorded in the Danish Central Husbandry Register (CHR) in 2016. This corresponds to nearly 2% of the total number of herds recorded in CHR that year. As shown in Table 1, the herd size of the organic herds is smaller than the conventional. Thus, the total number of organic pigs only corresponds to 0.8% of the total number of pigs recorded in CHR. Out of the 122 herds registered as organic pig herds in 2016, 57 had at least one antimicrobial prescription recorded in VetStat that year. Herds with antimicrobial prescriptions represent a larger proportion of all herds in conventional than in organic pig production (Table 1). A larger proportion of the antimicrobials for organic farms was handed over directly by the veterinarian (43% of the total amount of active compound) compared with the conventional farms (0.04%) which usually get the antimicrobials from the pharmacy (data not shown).

**Table 1:** Distribution of number of finishers and sows for all conventional and organic pig herds registered in CHR in 2016 and for conventional and organic pig herds registered in CHR and with antimicrobial prescription data in VetStat in 2016. The *p*-value reflects the statistical differences in number of animals for conventional and organic pig herds

	Herds in CHR						Herds in CHR with antimicrobial prescriptions					
	Number of sows^a^			Number of finishers^b^			Number of sows^a^			Number of finishers^b^		
	Conv. (*N* = 1949)	Org. (*N* = 73)	P-value	Conv. (*N* = 5556)	Org. (*N* = 102)	P-value	Conv. (*N* = 1755)	Organic (*N* = 29)	P-value	Conv. (*N* = 4819)	Organic (*N* = 51)	P-value
Min	1	1	*P* < 0.01	1	2	*P* < 0.01	1	3	*P* < 0.01	2	10	0.04
1st quartile	250	4		300	17		350	100		300	445	
Median	500	16		880	200		550	200		900	750	
3rd quartile	750	200		1600	750		800	360		1700	1465	
Max	3750	910		12,000	4000		3750	910		12,000	4000	

^a^In herds with > 1 sow in CHR; ^b^In herds with > 1 finisher in CHR

As shown in Table 2, the amount of antimicrobials prescribed for weaners was almost 14 times lower in organic than in conventional pig production, when measured in animal daily doses (ADD) per produced animal per year. For finishers, the amounts of antimicrobials prescribed were more than 4 times lower. Similar to prescriptions for conventional pig farms, gastro-intestinal indications also represented the most often used indication in organic pig farms. Compared with conventional production, a larger proportion was prescribed for respiratory indications in organic weaners and for arthropathy indications in organic finishers (Table 2).

**Table 2:** The number of ADD per produced animals per year for conventional and organic pig herds registered in CHR in 2016. The number of ADD for weaners and finishers are divided into the three predominant indications for which antimicrobials are prescribed in the Danish pig production

Age group	Indication	ADD/produced animals/year		Distribution per age group	
		Conventional	Organic	Conventional	Organic
Weaners (7–30 kg)	Gastro-intestinal	5.61	0.31	80%	65%
	Respiratory	0.68	0.15	10%	30%
	Arthropathy	0.72	0.03	10%	5%
	Total	7.01	0.49	100%	100%
Finishers (> 30 kg)	Gastro-intestinal	1.00	0.19	68%	54%
	Respiratory	0.15	0.02	10%	6%
	Arthropathy	0.32	0.14	22%	40%
	Total	1.47	0.35	100%	100%

**Fig. 1:**
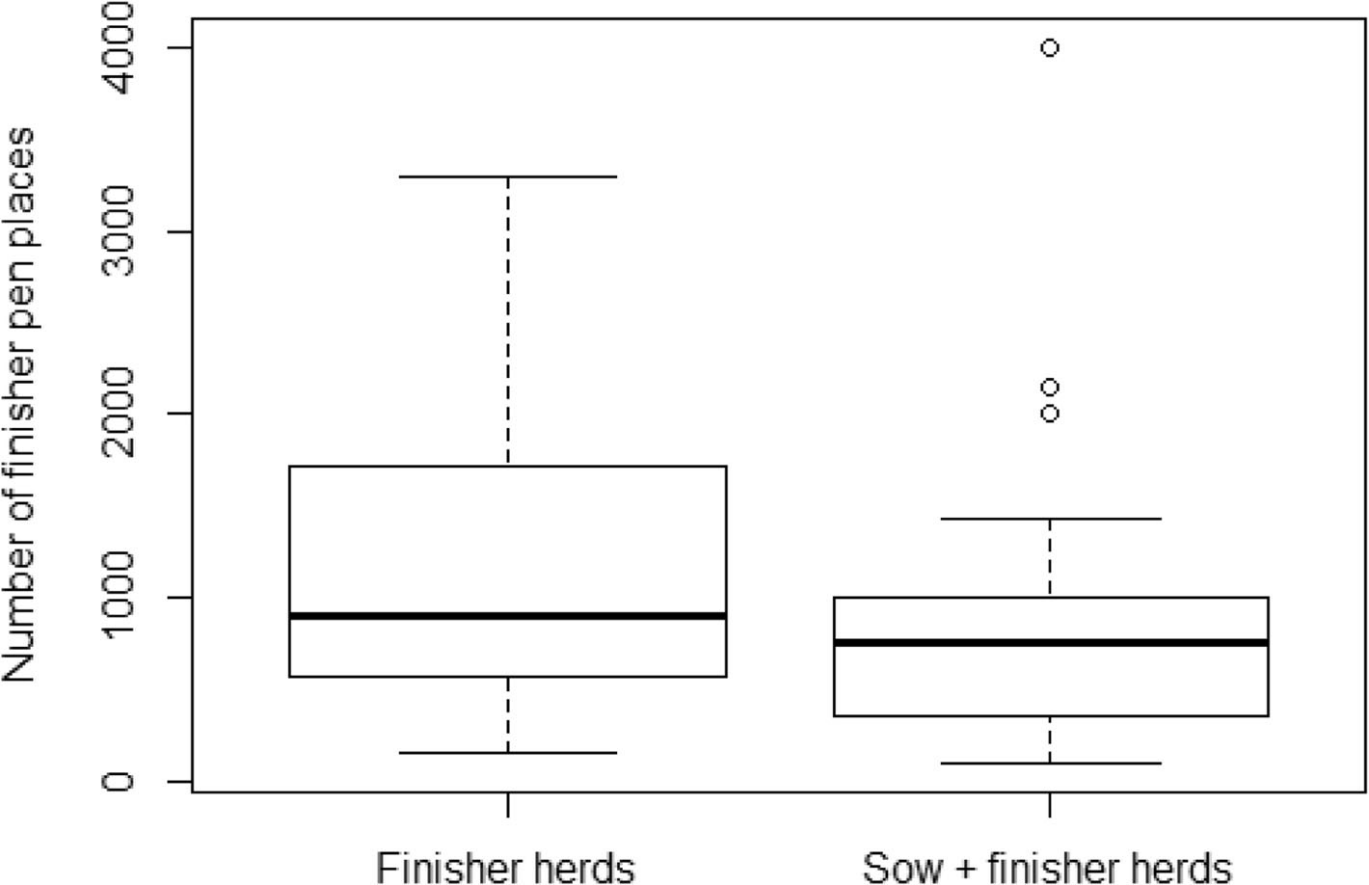
Boxplot showing the distribution of finisher pen places in organic finisher herds and sow + finisher herds, respectively

The second objective of this study was to study the associations between herd-level AMU prescription data and meat inspection data from organic herds. In total, 44 organic herds were included in this part of the study. The herds were grouped into finisher herds (*N* = 25) and sow + finisher herds (*N* = 19). The distribution of finisher pen places in the two groups is shown in Fig. 1.

The prevalences of lesions found as slaughter are presented in Table 3. A slightly higher occurrence of respiratory indications was seen in finisher herds (Table 3). However, the differences were non-significant.

**Table 3:** The prevalence of lesions at slaughter based on meat inspection data for organic sow + finisher herds and finisher herds with antimicrobials prescribed in 2016. The *p*-value reflects the statistical differences in prevalence of lesions at slaughter for the two types of herds

			Sow + finisher herds (*N* = 19)	Finisher herds (*N* = 25)	P-value
Prevalence of lesions at slaughter (%)	Gastrointestinal	Min	0.00	0.00	0.3
		1st quartile	0.00	0.00	
		Median	0.07	0.04	
		3rd quartile	0.10	0.09	
		Max	3.85	0.20	
	Respiratory	Min	1.37	3.51	0.3
		1st quartile	7.6	11.6	
		Median	11.9	23.9	
		3rd quartile	27.0	28.7	
		Max	47.8	59.3	
	Arthropathy	Min	0.17	0.05	0.2
		1st quartile	0.80	0.62	
		Median	1.55	1.01	
		3rd quartile	2.43	1.70	
		Max	14.8	2.94	

In total, 11 herds had no records of antimicrobials prescribed for finishers in 2016. Therefore, herds were divided into two groups; herds with AMU (*N* = 33) and without AMU (*N* = 11) for finishers in 2016. Herds without AMU registered for finishers had a slightly higher median prevalence of lesions at slaughter, specifically arthropathy lesions, than herds with AMU registered. A more similar distribution in the two groups was observed when considering the prevalence of gastro-intestinal and respiratory lesions (Table 4). However, the difference between the distributions for herds with and without AMU prescriptions in 2016 was not significant (for gastrointestinal: t = − 0.99, *p* = 0.4, for respiratory: t = 0.36, *p* = 0.7 and for arthropathy: t = − 1.12, *p* = 0.3).

**Table 4:** Prevalence of lesions found at slaughter in organic herds with and without AMU in 2016. The prevalence is presented for the three predominant indications in Danish pigs. The *p*-value reflects the statistical differences in prevalence of lesions at slaughter for herds with and without AMU

			Herds with AMU (*N* = 33)	Herds without AMU (*N* = 11)	P-value
Prevalence of lesions at slaughter (%)	Gastrointestinal	Min	0.00	0.00	0.4
		1st	0.00	0.00	
		Median	0.06	0.04	
		3rd	0.09	0.12	
		Max	0.20	3.85	
	Respiratory	Min	3.51	1.37	0.7
		1st	10.21	7.64	
		Median	14.50	18.52	
		3rd	28.68	29.14	
		Max	59.30	33.81	
	Arthropathy	Min	0.05	0.37	0.3
		1st	0.65	0.63	
		Median	1.09	1.25	
		3rd	1.73	2.44	
		Max	3.33	14.81	

**Fig. 2:**
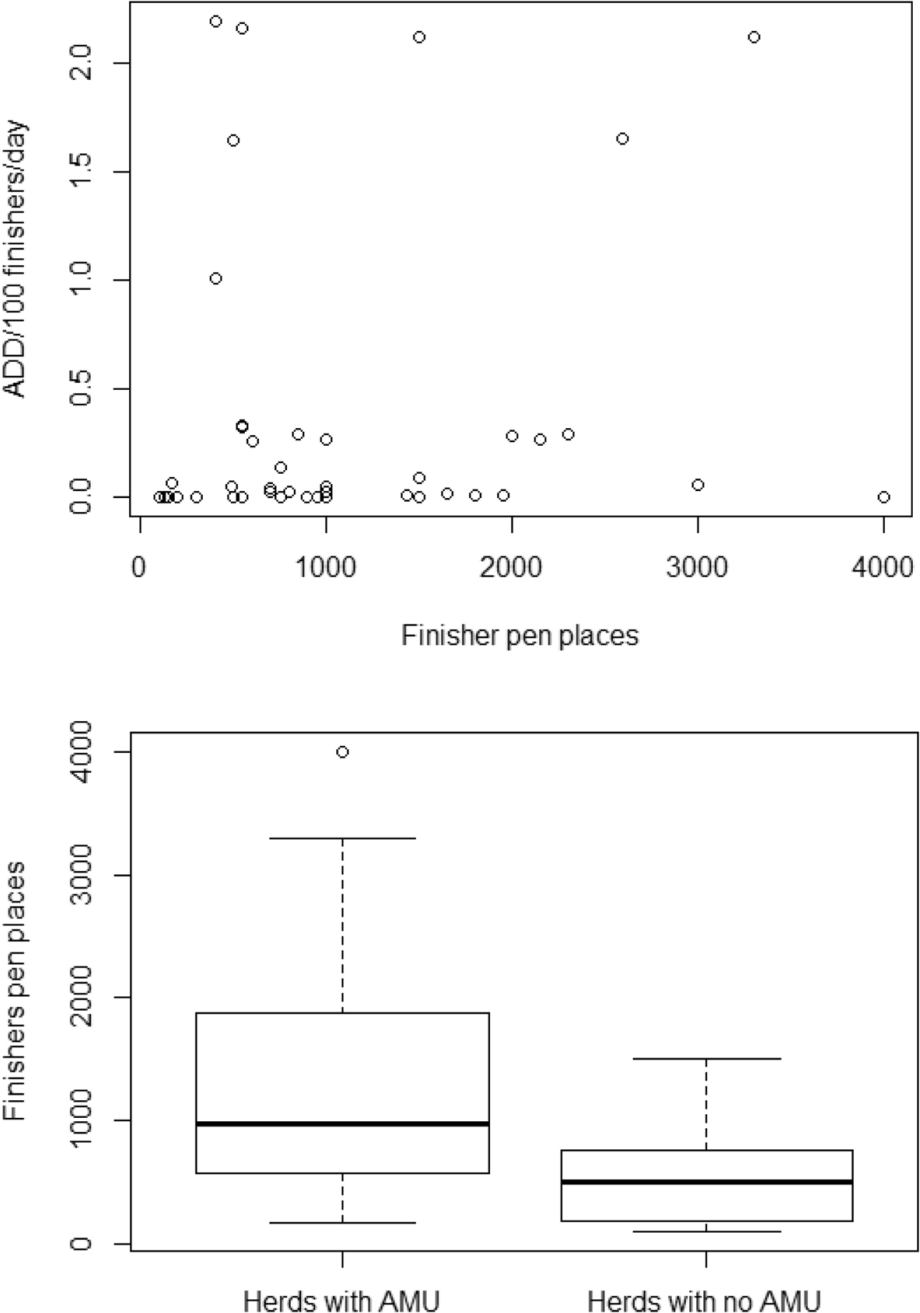
Scatterplot of AMU (measured in ADD/100 finishers/day) against number of finishers pen places. Boxplots showing the distribution of number of finisher pen places in organic pig herds with and without AMU in 2016

Herds with AMU had a significantly higher number of finishers (t = 3.55, *p* = 0.001) (Fig. 2).

The third objective of this study was to validate herd-level AMU prescription data in VetStat against treatment records collected on-farm in organic pig herds. Out of a target population of 54 organic pig herds, six herds were non-responders (could not be reached by telephone or e-mail). In total, farmers representing 17 herds were unwilling to participate due to time pressure (*N* = 7), no current production of pigs (*N* = 7) or due to personal reasons (*N* = 3). Finally, farmers representing 31 herds were willing to participate. Herd visits were performed in 20 herds and copies of treatment records were collected in each farm. Furthermore, treatment records were sent by e-mail from nine herds and finally two herds were interviewed by phone. However, for only eight herds in total, it was possible to collect sufficiently detailed data. The remaining herds were excluded since the AMU was registered as ml/kg, why the specific amounts used or the weights of the animals were not recorded. Thus, the presented results represent data from the remaining nine herds, for which their treatment records were compared to the prescription data from VetStat covering the same period. This comparison and the number of animals in each herd are shown in Table 5.

**Table 5:** Number of animals in the 8 herds participating in the study validating antimicrobial prescription data from VetStat with treatment records as primary data from 2016

Herd no.	Sows	Weaners	Finishers	AMU measured in mL		
				According to VetStat	According to Herd records	Difference
1	0	400	500	10,500	9135	1365 (13%)
2	385	110	950	5300	2755	2545 (48%)
3	400	9000	80	2700	1262	1438 (53%)
4	390	2000	150	305	672	− 367 (−120%)
5	0	1000	1500	42,001	38,418	3583 (9%)
6	100	246	585	900	120	780 (87%)
7	50	1900	2300	28,903	28,783	120 (0.4%)
8	550	1100	100	1600	188	1412 (88%)

Four ways of handling excess amount of antimicrobial products were identified based on information from the farmers. The most frequent way was to deposit it as hazardous waste (56%) and the next frequently used way was to return it to the veterinarian or pharmacies for destruction (33%). Finally, 11% of the farmers said the excess amounts were re-prescribed by their veterinarian.

## Discussion

The first part of the study showed that the prescribed AMU in organic pig herds is much lower than in conventional pig herds. Previous studies covering AMU in organic pig production is limited. For the present study, the use in weaners were 14 times lower, whereas for finisher pigs it was 4 times lower.

There might be different reasons for the low use. It might be due to the higher weaning age as suggested by Sjölund et al., [17]. In this study, a weaning age of 35 days were suggested as the reason for a lower AMU for weaners in Sweden. In Denmark, the organic pig production is obligated to have a higher weaning age than in conventional pig production (49 vs. 28 days) which might explain the lower use in organic pig production, and especially the larger difference between AMU for organic and conventional weaners than for finishers.

Furthermore, the withdrawal period from antimicrobial treatment to slaughter for organic pigs is twice the period for conventional pigs [3]. This means that there is a shorter period of the pigs live span where treatment is possible, which is also expected to contribute to a lower overall use, primarily in finishers.

For finisher herds, organic and conventional herds are more closely related than for sow herds, which might also explain why a smaller difference are seen between AMU for organic and conventional finishers than for weaners. Organic and conventional finisher pigs are often housed in similar stables. The main differences are that a fewer number of finishers per m^2^ are allowed in organic herds and that the animals need to have access to an outdoor area [4, 5]. These conditions might also contribute to the lower AMU seen in organic finishers.

In general, organic pig herds have fewer animals, which biologically would give a lower disease pressure, and again result in less infections and possibly also a lower need for AMU. Previous studies have also found a relationship between herd size and AMU in conventional pig herds (Kruse et al. 2017; [16]; Van der Fels-Klerx). This might not only reflect differences in disease pressure, but also different treatment and production strategies. Larger herds are often more productive, with an intensive production system, which often will require more treatment. This explanation might be applicable for both conventional and organic pig herds, since this study shows that organic herds with more finishers more often were herds with AMU.

The AMU might also be affected by official restriction on AMU. Despite restrictions on AMU in conventional pig herds, the restrictions for organic herds are much stricter. The veterinarian must always initiate antimicrobial treatment of animals in organic herds. After this, the veterinarian can prescribe antimicrobial for the rest of the treatment for maximum 5 days.

Pigs with a lifespan less than a year (weaners and finishers) are only allowed to be treated once in organic pig herds. If they need more than one treatment, they must be slaughtered as conventional pigs, and the farmer looses the supplement organic payment from the slaughterhouse. According to Alban et al. [2], this might give rise to a risk of under treatment, since farmers might me more reluctant to initiate treatment due to an economic incentive.

Pig raised free-range are expected to be challenged in different ways than indoor raised pigs, which can explain different disease patterns between organic and conventional pig production. This is also reflected in the prescription patterns presented in the first part of the study. For example, a higher proportion of AMU for respiratory indication in organic finishers, which might be explained by a lower use of vaccines against respiratory diseases in the organic pig production (VetStat data, unpublished). Alban et al., [1] showed that after introducing limits on the herd-level AMU in the Danish pig production in 2010 (The Yellow Card Initiative), the use of vaccines, especially targeting respiratory diseases increased in the conventional pig production. Looking at meat inspection data, a decline in respiratory findings were detected in the same period, and the increased use of vaccines were suggested as a possible explanation for that [1].

The second aim of this study was to compare prescription patterns and meat inspection at herd-level. A previous study on meat inspection data from organic and conventional herds, showed that three types of lesions were more often seen in organic pigs and free-range (old fractures, tail lesions and osteomyelitis) whereas four other types of lesions were more often seen in conventional pigs (abscess in leg/toe, hernia and scar/hock lesions). The number of lesions in total were equally distributed between production types. However, there were different disease problems occurring in the different types of production, which is probably a result of the different conditions and challenges indoor and outdoor, respectively [2].

No associations between the prescribed AMU and lesions were found, which might reflect that AMU might not be a good indication for disease status, at least not in herds with a low use. In Denmark, free-range (non-organic) production is similar to organic production, but less restricted on the AMU. The AMU is higher in free-range pig herds (VetStat data, unpublished) despite similar outdoor herd conditions. At least, these two types of production would be expected to have the same types of disease and therefore the same need for AMU. However, that is not reflected in the prescription data from VetStat.

Previous studies on the associations between AMU and lesions found at slaughter are limited, especially when studied at herd-level as in the present study. This might be due to a number of limitations with using meat inspection data. In Denmark, weak associations were found between routine meat inspection findings and results from systematic health monitoring when considering pericarditis, pleuritis and lung lesions [15]. Furthermore, some diseases, especially those occurring in the gastro-intestinal tract at earlier stage in life is difficult to detect at slaughter. Finally, animals that died on farm do not enter the slaughterhouse and therefore are not part of the statistics from the meat inspection.

In the third part of the study, we found that VetStat data is not a good proxy for AMU at herd-level in organic pig herds, and that this would most likely result in an overestimation. This was the case for 7 out of 8 herds. The farmers had different ways of handling the excess amount of antimicrobial products. Most alarming was that 11% of the farmers said the excess amounts were re-prescribed by the veterinarian. This is conflicting with the current legislation, requiring that the excess amount of antimicrobial product is discarded [3].

For one of the herds, the excess amount of antimicrobial products were negative, which means that the amount recorded in VetStat is actually lower than what is reported in the farm records. This reflects another problem with using VetStat for estimating AMU in organic herds. Organic herds often gets their medicine directly from their veterinarian. When antimicrobials are sold from the pharmacies (still with a prescription), the receiving herd is automatically registered. When the product are handed over directly from the veterinarian, it requires that the veterinarian report for which CHR number the products are prescribed for. If not, this will result in an under-estimation of the actual use for that prescription.

Many errors in the treatment records were detected, making it difficult to compare with data from VetStat. A total of 22 herds were excluded from the study due to incorrect treatment records. This is an interesting finding, since it might reflect a general problem with registering the AMU despite a legal obligation to do so. However, problems with AMU registration at herd-level is not only seen in organic pig production, but also in the conventional pig production. A campaign by the Danish Veterinary and Food Administration (DVFA) in a subset of Danish conventional finisher herds revealed that in 19% of the cases, the farmer did not report all antimicrobial treatments or did not follow the instructions given by the veterinarian in relation to dosage or indication [9].

Excluding a large proportion of the herds also resulted in a small sample of herds with a large variation in differences between VetStat and treatment records. This makes it difficult to extrapolate these results. It would be of relevance to consider this as a focus point in future campaigns by the Veterinary Authorities. In addition, when working with AMU data from organic pig herds from VetStat, researchers should make interpretations with these results in mind.

## Conclusions

Compared with conventional production,the prescribed AMU in organic pig herds is 14 times lower for weaners, and 4 times lower for finishers. Furthermore, a larger proportion of AMU is prescribed for respiratory indications in organic weaners and arthropathy indications in organic finishers. No clear association between the AMU and lesions at slaughter were found. However, prevalence of lesions was slightly (non-significant) higher in herds with no registered AMU than with AMU prescriptions. Discrepancies were seen when comparing AMU registered in VetStat and herd treatment records in organic farms. Many errors in the treatment records in organic farms were detected, making it difficult to compare with data from VetStat.

## Methods

Prescription data from 2016 originating from two Danish databases were used in all three parts of the study. Firstly, herd-level antimicrobial prescription data were extracted from VetStat. These data were used as a proxy for AMU in this study. Each prescription was converted into milligram active compound and animal daily dose (ADD), based on a list for each product in VetStat, provided by the Danish Veterinary and Food Administration (DVFA). In Vetstat, the indication for which each product is prescribed is given. Based on this information the AMU for the three predominant indications used in both conventional and organic pig production were summarised. These were gastro-intestinal, respiratory and arthropathy indications.

Finally, information regarding herd type and number of animals in each herd were extracted from CHR.

Both the CHR and VetStat databases are owned and maintained by the DVFA.

In the first part of the study, all herds registered as either organic or conventional pig herds in 2016 were included. These herds were grouped according to herd type, and the two types of herds were compared descriptively in terms of antimicrobial use and herd size.

The AMU measured in ADDs were divided by the number of animals produced in 2016. These numbers were based on slaughterhouse statistics for conventional and organic pig production, respectively.

For the second part of the study, all organic herds fulfilling the following enrolment criteria were included: Herds with either; 1) No sows and > 150 finisher (finisher herds) or 2) Sows > 50 and finisher > 150 (sow + finisher herds). By using the number of pen places in CHR, it was possible to calculate the average ADD/100 finishers/day in 2016. Furthermore, herds with meat inspection data available were included.

Meat inspection data were obtained from the Danish slaughterhouse database. A criterion of a minimum slaughter weight at 110 k was set to only included finisher pigs sent for slaughter. Based on these data, the number of pigs produced in each herd were found. Based on the codes in the meat inspection data, lesions found at slaughter were divided into three main groups of lesions as previous described [13]. These three groups were gastro-intestinal (acute/chronic gastritis, acute/chronic enteritis, serosal adhesions, stomach ulcer or rectal stricture), respiratory (sinuitis/rhinitis, acute/chronic pneumonia or acute/chronic pleuritis) and arthropathy (acute/chronic arthritis).

For many of the organic herds, the ADD/100 finishers/day recorded in VetStat were zero. Therefore, it was impossible to consider the ADD/100 finisher/day as a continuous variable in the analysis. Instead, herds were divided into two groups; herds with and without AMU in 2016. Patterns in lesions found at slaughter for the two groups were evaluated descriptively and the differences were analysed with student’s t-tests using the software R, version 3.3.2.

The organic herds included in the third part of the study were also herds with either; 1) No sows and > 150 finisher (finisher herds) or 2) Sows > 50 and > 150 (sow + finishers herds). All herd owners were contacted through e-mail and telephone for participation. For farmers willing to participate, a herd visit was scheduled. At the herd visits, primary data consisting of the treatment records and information on handling of excess amount of antimicrobial products were collected. In order for a herds to have sufficiently detailed data, the farmer had registered the actual amount used for a treatment, the duration, and the weight of the animal treated. Herds with sufficiently detailed data were included in the calculations of excess antimicrobial products. This was calculated as the difference between the products prescribed in VetStat and the products used according to the treatment records.

## Abbreviations

ADD: Animal daily doses
AMR: Antimicrobial resistance
AMU: Antimicrobial use
CHR: Central husbandry register
DVFA: Danish veterinary and food administration
